# Real-world patient characteristics and treatment patterns in US patients with advanced non-small cell lung cancer

**DOI:** 10.1186/s12885-024-12126-8

**Published:** 2024-04-06

**Authors:** Hozefa A. Divan, Marisa A. Bittoni, Ashok Krishna, David P. Carbone

**Affiliations:** 1 Sanofi, Inc., 450 Water Street, Cambridge, MA 02142 USA; 2 James Comprehensive Cancer Center, The Ohio State University, 460 West 10th Avenue, Columbus, OH 43210 USA

**Keywords:** Non-small cell lung cancer, PD-1 inhibitors, PD-L1 inhibitors, Real-world evidence, Patient characteristics, Observational study

## Abstract

**Background:**

Patients from non-small cell lung cancer (NSCLC) controlled clinical trials do not always reflect real-world heterogeneous patient populations. We designed a study to describe the real-world patient characteristics and treatment patterns of first-line treatment in patients in the US with NSCLC.

**Methods:**

This was an observational, retrospective cohort study based on electronic medical records of US adults with locally advanced or metastatic disease in the ConcertAI Patient360 NSCLC database who initiated first-line treatment with anti-programmed cell death protein 1/programmed cell death ligand 1 (PD-1/PD-L1) therapy between July 2016 and December 2020. The analysis used patient attributes, clinical characteristics, and treatments from each patient’s medical records.

**Results:**

A total of 2175 patients were eligible for analysis. The median age was 68 years, and 26.2% of the patients were ≥75 years old. At treatment initiation, 96.4% and 3.6% of the patients had Stage 4 and Stage 3 (B or C) NSCLC, respectively. The most common histology type was nonsquamous adenocarcinoma (66.4%), and 19.8% had Eastern Cooperative Oncology Group performance status ≥2. Immunosuppressive medications were being used by 17.7% of patients, and 11.0% were immunocompromised. Almost all patients had metastases: 64.6% had 1, 23.2% had 2, and 8.0% had ≥3 metastatic sites. Brain metastases were present in 22.9% of patients. Treatment evolution was observed with first-line standard of care shifting from single-agent immunotherapy in 2016 (90.2%) to combination immunotherapy and chemotherapy in 2020 (60.2%).

**Conclusion:**

Between 2016 and 2020, the first-line treatment paradigm for advanced NSCLC in the US shifted from anti–PD-1/PD-L1 monotherapy to combination chemoimmunotherapy, with increasing biomarker testing. Further research in heterogeneous patient populations to characterize treatment strategies is warranted.

## Background

Lung cancer was the leading cause of cancer-related deaths worldwide in 2020, and approximately 70% of cases were locally advanced or metastatic disease at diagnosis, of which 80–85% were non-small cell lung cancer (NSCLC) [1–3].

Clinical trials in recent years have examined a variety of treatment strategies, including monotherapy and combination immunotherapy (IO) compared with chemo-therapy, the previous standard of care for advanced NSCLC [4–15]. As a result, the treatment landscape for advanced NSCLC without actionable driver mutations in *EGFR* or *ALK* has shifted from chemotherapy to IO with chemotherapy. Immunotherapies target negative immunologic regulators such as cytotoxic T lymphocyte-associated antigen 4 (CTLA-4) and the programmed cell death protein 1 (PD-1)/programmed cell death ligand 1 (PD-L1) pathway [16].

Between 2015 and 2021, IO agents approved by the United States (US) Food and Drug Administration (FDA) as first- or second-line therapy for NSCLC without driver mutations included: the anti–PD-1 antibodies nivolumab, pembrolizumab, and cemiplimab; the anti–PD-L1 antibodies durvalumab and atezolizumab; and the anti–CTLA-4 antibody ipilimumab [17]. Use of first-line IO to treat advanced NSCLC has increased substantially in the US since the initial approvals in 2016 [18]. First-line FDA approvals occurred for IO monotherapy with pembrolizumab in October 2016 and for pembrolizumab combination therapy with chemotherapy in May 2017 [19]. Atezolizumab combination therapy was approved in December 2018 [19]. Nivolumab + ipilimumab was approved in May 2020, along with atezolizumab monotherapy [19]. Finally, cemiplimab monotherapy was approved in February 2021 [19].

Clinical trials are designed to enroll selected patients (ie, those with good performance status, adequate organ function, without certain comorbidities, and who are not immunocompromised), and treatments are administered in highly controlled settings. Therefore, it can be challenging to generalize the findings to the more clinically heterogeneous patient populations seen in practice [18]. Here we report the findings from a real-world observational study that examined the ConcertAI Patient360 NSCLC database to describe key evidence gaps related to clinical characteristics and treatment patterns in patients in the US who initiated first-line treatment with IO mono-therapy or combination therapy for advanced NSCLC from 2016 to 2020.

## Methods

### Study design and data source

This was a non-interventional, observational, retrospective cohort study of patients with advanced NSCLC who received treatment as documented in the Patient360 NSCLC electronic medical record (EMR) database (ConcertAI, Cambridge, MA). This database sources patient EMRs, including unstructured notes and scans, from multiple oncologic partnerships that are EMR-system agnostic. The database consists of de-identified data from patients treated at various academic (~20%) and community (~80%) oncology centers across the US (~15% in the Northeast, ~25% in the Midwest, ~40% in the South, and ~20% in the West, as defined by US Census Bureau geographic regions).

**Fig. 1:**
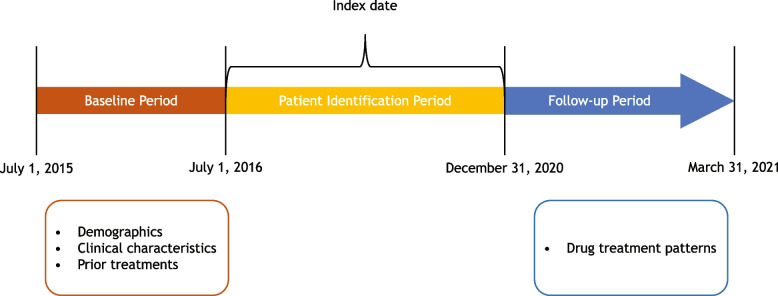
Study period timeline, including baseline period, patient identification period, index date, and follow-up period

The overall study period was from July 1, 2015, to March 31, 2021 (Fig. 1). The study design included a baseline period, a patient identification period, and a follow-up period. The index date was defined as the date on which the patient initiated first-line anti–PD-1/PD-L1 therapy for locally advanced or metastatic NSCLC. The baseline period spanned from the patient’s earliest NSCLC diagnosis in the database, starting from July 1, 2015, to the index date. If more than one assessment for the same variable of interest was available within this baseline period, the assessment closest to the index date was selected. During the patient identification period (July 1, 2016, to December 31, 2020; 3 months prior to the end of the follow-up period), eligible patients with advanced NSCLC who initiated first-line systemic treatment were identified. The follow-up period began one day post-index date and ended either on the date of death or on March 31, 2021 (end date of the database).

### Patients

**Fig. 2:**
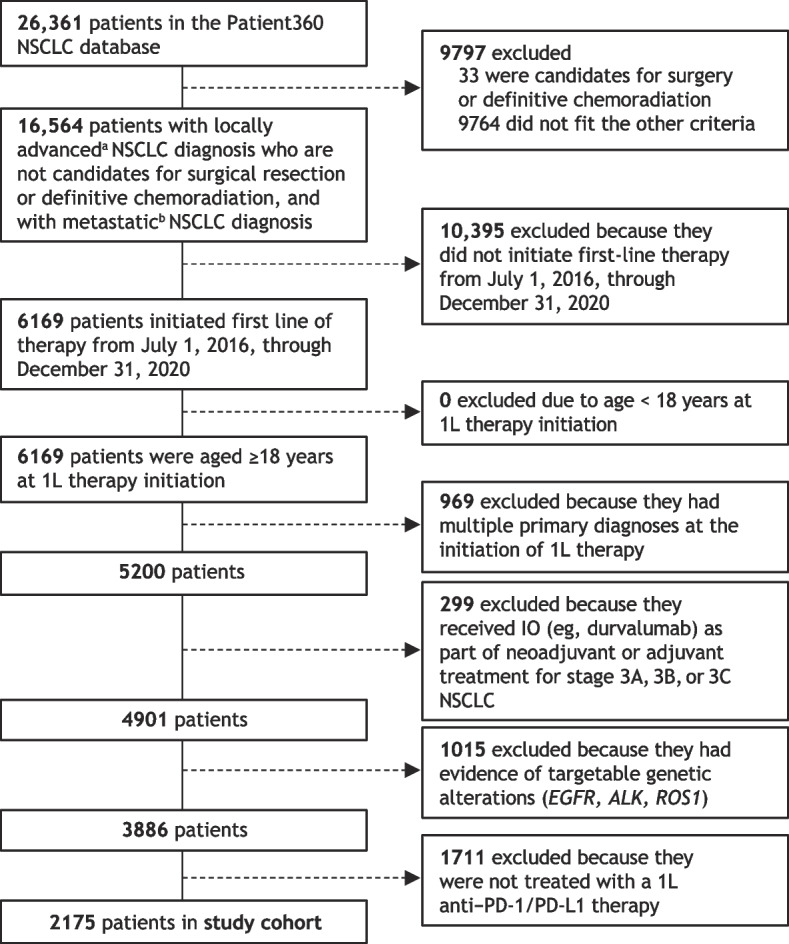
Patient selection and study cohort derived from the Patient360 NSCLC database. 1L, first line; *ALK*, anaplastic lymphoma kinase; *EGFR*, epidermal growth factor receptor; IO, immunotherapy; NSCLC, non-small cell lung cancer; PD-1, programmed cell death protein 1; PD-L1, programmed cell death ligand 1; *ROS1*, ROS proto-oncogene 1, receptor tyrosine kinase. ^a^Stage 3 (3B, 3C) or neoplasm, secondary; ^b^Stage 4, M1 or neoplasm, metastatic

The focus of this analysis was patients with advanced NSCLC who were treated with first-line anti–PD-1/PD-L1 therapy. Patients included in the final study cohort for this analysis (Fig. 2) had to have (i) a diagnosis of locally advanced, unresectable Stage 3B and 3C, or Stage 4 metastatic NSCLC; (ii) no evidence of candidacy for surgical reconstruction or definitive chemoradiation; (iii) started first-line therapy from July 1, 2016, through December 3, 2020; (iv) age ≥18 years at the time of first-line therapy initiation; (v) absence of a multiple primary cancer diagnosis at the time of initiating first-line therapy; (vi) no evidence of IO treatment in Stage 3 (A, B, or C) NSCLC as part of neoadjuvant or adjuvant treatment; (vii) no evidence of targetable genetic alterations (eg, *EGFR*, *ALK*, *ROS1*); and (viii) received first-line treatment with an anti–PD-1/PD-L1 agent for NSCLC.

### Study objectives

The study objectives were to describe the demographics, clinical characteristics, and drug treatment patterns in patients with advanced NSCLC who were treated with first-line anti–PD-1/PD-L1 therapy. This was done for the overall cohort and by year of initiation of first-line therapy (2016 to 2020) and stratified by subgroups of interest that represent clinically relevant, unmet-needs populations. Additionally, clinical characteristics were stratified by first-line therapy, ie, anti–PD-1/PD-L1 monotherapy or anti–PD-1/PD-L1 therapy combined with platinum-based chemotherapy.

### Ethical considerations

The study was performed in accordance with the Declaration of Helsinki and relevant International Council for Harmonisation, Good Clinical Practice, and Good Pharmacoepidemiological Practice guidelines. As no identifiable protected health information was extracted or accessed for the conduct of this study, ethics approval was deemed unnecessary under the Code of Federal Regulations Title 45, Part 46, Section 46.104(d)(4)(ii) (45CFR46.104[d][4][ii]).

### Statistical analysis

This was an observational, descriptive cohort study of real-world patients with advanced NSCLC; hence, no hypothesis was tested, and no formal sample size calculation was required. Study measures (ie, patient demographics, disease and clinical characteristics, recorded treatment) were summarized with descriptive statistics and presented as frequencies and percentages. Statistical analyses were conducted using the Palantir Foundry implementation of PySpark.

## Results

### Cohort disposition

From an initial population of 26,361 curated patients available in the NSCLC database, 3886 patients met all criteria and were started on first-line therapy in the study period, and 2175 of these were treated with an anti–PD-1/PD-L1 agent in the first line during the specified period and were included in the study cohort (Fig. 2).

### Patient characteristics

Baseline demographics and clinical characteristics of the patients in the study cohort (*n* = 2175) are summarized in Table 1, overall and by year of first-line therapy initiation. In this eligible population, the median age was 68.0 years (range, 19.0 to 88.0 years), and 53.7% were male. The age group distribution of the cohort remained constant from 2016 to 2020. Most patients were White (76.8%); African Americans comprised the second largest racial group (13.2%). Current or former smokers made up 87.9% of study patients, with the proportion increasing from 83.3% of those who initiated first-line therapy in 2016 to 92.9% of those who initiated first-line therapy in 2020.

**Table 1:** Baseline demographic and clinical characteristics of patients overall and by initiation of first-line therapy (year)

**Parameters at index date**	**Eligible population *N* = 2175**	**Index date (year) of first line of therapy**						
		**2016, *n* = 102**	**2017, *n* = 376**	**2018, *n* = 586**	**2019, *n* = 717**	**2020, *n* = 394**	**2016 to 2017, *n* = 478**	**2018 to 2020, *n* = 1697**
**Median age (range), years**	68.0 (19.0, 88.0)	68.0 (37.0, 84.0)	68.0 (26.0, 85.0)	68.0 (24.0, 86.0)	67.0 (19.0, 87.0)	68.0 (40.0, 88.0)	68.0 (26.0, 85.0)	68.0 (19.0, 88.0)
**Age group, years, n (%)**								
<65	846 (38.9)	37 (36.3)	148 (39.4)	222 (37.9)	298 (41.6)	141 (35.8)	185 (38.7)	661 (39.0)
≥65 to <75	759 (34.9)	38 (37.3)	128 (34.0)	205 (35.0)	238 (33.2)	150 (38.1)	166 (34.7)	593 (34.9)
≥75	570 (26.2)	27 (26.5)	100 (26.6)	159 (27.1)	181 (25.2)	103 (26.1)	127 (26.6)	443 (26.1)
**Sex, n (%)**								
Male	1169 (53.7)	43 (42.2)	207 (55.1)	305 (52.0)	404 (56.3)	210 (53.3)	250 (52.3)	919 (54.2)
Female	1006 (46.3)	59 (57.8)	169 (44.9)	281 (48.0)	313 (43.7)	184 (46.7)	228 (47.7)	778 (45.8)
**Race, n (%)**								
White	1670 (76.8)	82 (80.4)	283 (75.3)	444 (75.8)	563 (78.5)	298 (75.6)	365 (76.4)	1305 (76.9)
Black or African American	288 (13.2)	16 (15.7)	56 (14.9)	69 (11.8)	92 (12.8)	55 (14.0)	72 (15.1)	216 (12.7)
Asian	53 (2.4)	1 (1.0)	11 (2.9)	22 (3.8)	12 (1.7)	7 (1.8)	12 (2.5)	41 (2.4)
American Indian or Alaska Native	24 (1.1)	0 (0)	3 (0.8)	6 (1.0)	9 (1.3)	6 (1.5)	3 (0.6)	21 (1.2)
Native Hawaiian or Other Pacific Islander	2 (0.1)	0 (0)	0 (0)	1 (0.2)	1 (0.1)	0 (0)	0 (0)	2 (0.1)
Other or Unknown Race	138 (6.3)	3 (2.9)	23 (6.1)	44 (7.5)	40 (5.6)	28 (7.1)	26 (5.4)	112 (6.6)
**Ethnicity, n (%)**								
Hispanic or Latino	50 (2.3)	0 (0)	9 (2.4)	14 (2.4)	17 (2.4)	10 (2.5)	9 (1.9)	41 (2.4)
Not Hispanic or Latino	1924 (88.5)	88 (86.3)	333 (88.6)	523 (89.2)	634 (88.4)	346 (87.8)	421 (88.1)	1503 (88.6)
Unknown	201 (9.2)	14 (13.7)	34 (9.0)	49 (8.4)	66 (9.2)	38 (9.6)	48 (10.0)	153 (9.0)
**Smoking status, n (%)**								
Yes (current or former smoker)	1912 (87.9)	85 (83.3)	313 (83.2)	501 (85.5)	647 (90.2)	366 (92.9)	398 (83.3)	1514 (89.2)
No (never smoker)	263 (12.1)	17 (16.7)	63 (16.8)	85 (14.5)	70 (9.8)	28 (7.1)	80 (16.7)	183 (10.8)
**Histology, n (%)**								
Nonsquamous	1447 (66.5)	65 (63.7)	256 (68.1)	400 (68.3)	450 (62.8)	276 (70.1)	321 (67.2)	1126 (66.4)
Adenocarcinoma only	1445 (66.4)	64 (62.7)	251 (66.8)	403 (68.8)	454 (63.3)	273 (69.3)	315 (65.9)	1130 (66.6)
Squamous	457 (21.0)	24 (23.5)	70 (18.6)	120 (20.5)	167 (23.3)	76 (19.3)	94 (19.7)	363 (21.4)
Adeno-squamous carcinoma	34 (1.6)	0 (0)	3 (0.8)	12 (2.0)	17 (2.4)	2 (0.5)	3 (0.6)	31 (1.8)
NSCLC NOS	152 (7.0)	9 (8.8)	38 (10.1)	32 (5.5)	51 (7.1)	22 (5.6)	47 (9.8)	105 (6.2)
**Stage, n (%)**								
3	78 (3.6)	3 (2.9)	15 (4.0)	19 (3.2)	26 (3.6)	15 (3.8)	18 (3.8)	60 (3.5)
4	2097 (96.4)	99 (97.1)	361 (96.0)	567 (96.8)	691 (96.4)	379 (96.2)	460 (96.2)	1637 (96.5)
**Number of metastatic sites, n (%)**								
0	23 (1.1)	2 (2.0)	4 (1.1)	6 (1.0)	7 (1.0)	4 (1.0)	6 (1.3)	17 (1.0)
1	1404 (64.6)	64 (62.7)	239 (63.6)	361 (61.6)	443 (61.8)	297 (75.4)	303 (63.4)	1101 (64.9)
2	504 (23.2)	22 (21.6)	90 (23.9)	149 (25.4)	173 (24.1)	70 (17.8)	112 (23.4)	392 (23.1)
≥3	173 (8.0)	8 (7.8)	32 (8.5)	50 (8.5)	69 (9.6)	14 (3.6)	40 (8.4)	133 (7.8)
Missing	71 (3.3)	6 (5.9)	11 (2.9)	20 (3.4)	25 (3.5)	9 (2.3)	17 (3.6)	54 (3.2)
**Location of metastases, n (%)**								
Lung	433 (19.9)	24 (23.5)	81 (21.5)	107 (18.3)	141 (19.7)	80 (20.3)	105 (22.0)	328 (19.3)
Brain	499 (22.9)	18 (17.6)	81 (21.5)	156 (26.6)	163 (22.7)	81 (20.6)	99 (20.7)	400 (23.6)
Bone	649 (29.8)	23 (22.5)	113 (30.1)	188 (32.1)	228 (31.8)	97 (24.6)	136 (28.5)	513 (30.2)
Liver	289 (13.3)	15 (14.7)	49 (13.0)	72 (12.3)	106 (14.8)	47 (11.9)	64 (13.4)	225 (13.3)
Skin	11 (0.5)	2 (2.0)	2 (0.5)	1 (0.2)	3 (0.4)	3 (0.8)	4 (0.8)	7 (0.4)
Lymph nodes	223 (10.3)	11 (10.8)	44 (11.7)	62 (10.6)	77 (10.7)	29 (7.4)	55 (11.5)	168 (9.9)
Other	883 (40.6)	41 (40.2)	150 (39.9)	243 (41.5)	304 (42.4)	145 (36.8)	191 (40.0)	692 (40.8)
None	23 (1.1)	2 (2.0)	4 (1.1)	6 (1.0)	7 (1.0)	4 (1.0)	6 (1.3)	17 (1.0)
Missing	71 (3.3)	6 (5.9)	11 (2.9)	20 (3.4)	25 (3.5)	9 (2.3)	17 (3.6)	54 (3.2)
**Evidence of visceral metastasis**^**a**^**, n (%)**								
Present	1716 (78.9)	80 (78.4)	301 (80.1)	463 (79.0)	568 (79.2)	304 (77.2)	381 (79.7)	1335 (78.7)
Absent	459 (21.1)	22 (21.6)	75 (19.9)	123 (21.0)	149 (20.8)	90 (22.8)	97 (20.3)	362 (21.3)
**Evidence of nonvisceral metastasis, n (%)**								
Present	817 (37.6)	33 (32.4)	143 (38.0)	232 (39.6)	283 (39.5)	126 (32.0)	176 (36.8)	641 (37.8)
Absent	1358 (62.4)	69 (67.6)	233 (62.0)	354 (60.4)	434 (60.5)	268 (68.0)	302 (63.2)	1056 (62.2)
**ECOG PS groups, n (%)**								
0 or 1	1408 (64.7)	66 (64.7)	212 (56.4)	358 (61.1)	486 (67.8)	286 (72.6)	278 (58.2)	1130 (66.6)
2+	430 (19.8)	23 (22.5)	87 (23.1)	105 (17.9)	131 (18.3)	84 (21.3)	110 (23.0)	320 (18.9)
Unknown	337 (15.5)	13 (12.7)	77 (20.5)	123 (21.0)	100 (13.9)	24 (6.1)	90 (18.8)	247 (14.6)
**Patients by recorded line of therapy, n (%)**								
1	2175 (100.0)	102 (100.0)	376 (100.0)	586 (100.0)	717 (100.0)	394 (100.0)	478 (100.0)	1697 (100.0)
2	531 (24.4)	27 (26.5)	124 (33.0)	170 (29.0)	158 (22.0)	52 (13.2)	151 (31.6)	380 (22.4)
3	136 (6.3)	7 (6.9)	47 (12.5)	48 (8.2)	31 (4.3)	3 (0.8)	54 (11.3)	82 (4.8)
≥4	38 (1.7)	1 (1.0)	18 (4.8)	13 (2.2)	6 (0.8)	0 (0)	19 (4.0)	19 (1.1)
**History of autoimmune disease, n (%)**								
Yes	33 (1.5)	2 (2.0)	7 (1.9)	8 (1.4)	9 (1.3)	7 (1.8)	9 (1.9)	24 (1.4)
No	2142 (98.5)	100 (98.0)	369 (98.1)	578 (98.6)	708 (98.7)	387 (98.2)	469 (98.1)	1673 (98.6)
**Patient Immunocompromised, n (%)**^**b,c**^								
Yes	240 (11.0)	16 (15.7)	46 (12.2)	63 (10.8)	73 (10.2)	42 (10.7)	62 (13.0)	178 (10.5)
No	1935 (89.0)	86 (84.3)	330 (87.8)	523 (89.2)	644 (89.8)	352 (89.3)	416 (87.0)	1519 (89.5)
**Use of immunosuppressive medication, n (%)**								
Any								
Yes	385 (17.7)	26 (25.5)	72 (19.1)	109 (18.6)	125 (17.4)	53 (13.5)	98 (20.5)	287 (16.9)
No	1790 (82.3)	76 (74.5)	304 (80.9)	477 (81.4)	592 (82.6)	341 (86.5)	380 (79.5)	1410 (83.1)
Prednisone/prednisolone								
Yes	367 (16.9)	25 (24.5)	71 (18.9)	106 (18.1)	116 (16.2)	49 (12.4)	96 (20.1)	271 (16.0)
Short-term use (<30 days)	200 (9.2)	13 (12.7)	39 (10.4)	64 (10.9)	69 (9.6)	15 (3.8)	52 (10.9)	148 (8.7)
Long-term use (≥30 days)	225 (10.3)	16 (15.7)	45 (12.0)	61 (10.4)	65 (9.1)	38 (9.6)	61 (12.8)	164 (9.7)
No	18 (0.8)	1 (1.0)	1 (0.3)	3 (0.5)	9 (1.3)	4 (1.0)	2 (0.4)	16 (0.9)
Other immunosuppressive medications								
Yes	35 (1.6)	2 (2.0)	4 (1.1)	6 (1.0)	13 (1.8)	10 (2.5)	6 (1.3)	29 (1.7)
Short-term use (<30 days)	8 (0.4)	0 (0)	0 (0)	1 (0.2)	7 (1.0)	0 (0)	0 (0)	8 (0.5)
Long-term use (≥30 days)	23 (1.1)	1 (1.0)	4 (1.1)	4 (0.7)	6 (0.8)	8 (2.0)	5 (1.0)	18 (1.1)
No	350 (16.1)	24 (23.5)	68 (18.1)	103 (17.6)	112 (15.6)	43 (10.9)	92 (19.2)	258 (15.2)
***RET* test recorded, n (%)**	1076 (49.5)	39 (38.2)	146 (38.8)	242 (41.3)	355 (49.5)	294 (74.6)	185 (38.7)	891 (52.5)
Positive	73 (3.4)	6 (5.9)	10 (2.7)	16 (2.7)	17 (2.4)	24 (6.1)	16 (3.3)	57 (3.4)
***BRAF* test recorded, n (%)**	1280 (58.9)	39 (38.2)	171 (45.5)	320 (54.6)	440 (61.4)	310 (78.7)	210 (43.9)	1070 (63.1)
Positive	108 (5.0)	5 (4.9)	15 (4.0)	25 (4.3)	37 (5.2)	26 (6.6)	20 (4.2)	88 (5.2)
***MET* exon 14 skipping mutation test, n (%)**	1062 (48.8)	36 (35.3)	142 (37.8)	236 (40.3)	354 (49.4)	294 (74.6)	178 (37.2)	884 (52.1)
Positive	0	0	0	0	0	0	0	0
***RET, BRAF,* or *MET* test recorded, n (%)**	1307 (60.1)	40 (39.2)	178 (47.3)	330 (56.3)	447 (62.3)	312 (79.2)	218 (45.6)	1089 (64.2)
Positive	179 (8.2)	10 (9.8)	25 (6.6)	41 (7.0)	53 (7.4)	50 (12.7)	35 (7.3)	144 (8.5)
**PD-1/PD-L1 test recorded, n (%)**	1566 (72.0)	55 (53.9)	258 (68.6)	424 (72.4)	517 (72.1)	312 (79.2)	313 (65.5)	1253 (73.8)
Unknown	90 (4.1)	1 (1.0)	23 (6.1)	38 (6.5)	25 (3.5)	3 (0.8)	24 (5.0)	66 (3.9)
Negative	498 (22.9)	20 (19.6)	72 (19.1)	124 (21.2)	159 (22.2)	123 (31.2)	92 (19.2)	406 (23.9)
Positive	978 (45.0)	34 (33.3)	163 (43.4)	262 (44.7)	333 (46.4)	186 (47.2)	197 (41.2)	781 (46.0)
**PD-1/PD-L1 expression level, n (%)**								
PD-1/PD-L1 expression 1–49%^d^	62 (2.9)	2 (2.0)	17 (4.5)	21 (3.6)	19 (2.6)	3 (0.8)	19 (4.0)	43 (2.5)
PD-1/PD-L1 expression ≥50%^d^	115 (5.3)	4 (3.9)	26 (6.9)	36 (6.1)	43 (6.0)	6 (1.5)	30 (6.3)	85 (5.0)

*BRAF* proto-oncogene B-Raf, *ECOG PS* Eastern Cooperative Oncology Group performance status, *HIV* human immunodeficiency virus, *MET* mesenchymal epithelial transition factor, *NOS* not otherwise specified, *NSCLC* non-small cell lung cancer, *PD-1* programmed cell death protein 1, *PD-L1* programmed cell death ligand 1, *RET* RET proto-oncogene

^a^Pulmonary (lung), hepatic (liver), pleural, pleural effusions, peritoneal and ascites involvement. Patients with visceral metastases irrespective of the presence of any other metastatic sites (eg, bone) can be categorized as visceral. All other patients without visceral metastases can be categorized as nonvisceral

^b^Immunocompromised defined as having HIV or taking long-term (≥30 days) immunosuppressive medications

^c^Having HIV, long-term use of prednisone/prednisolone, and long-term use of other immunosuppressive medications are not mutually exclusive

^d^At the time of analysis, the completeness/availability of expression results in the Patient360 NSCLC database was limited

Approximately two-thirds of patients (66.4%) had nonsquamous NSCLC with adenocarcinoma histology (Table 1). Nearly all patients (96.4%) had Stage 4 disease, and 95.7% had one or more metastatic sites. Bone (29.8%), brain (22.9%), and other lung (19.9%) were the most common metastatic sites at the index date. More patients younger than 65 years (29.3%) had evidence of brain metastases than those aged 65–74 years (21.7%) and ≥75 years (15.1%) (Table 2). More than three-quarters (78.9%) of patients had visceral site(s) of metastases at the index date, and 37.6% had nonvisceral site(s) of metastases (Table 1). Almost two-thirds of patients (64.7%) with reported Eastern Cooperative Oncology Group performance status (ECOG PS) had a score of 0 or 1, and 19.8% had a score of 2 or higher.

**Table 2:** Baseline demographic, clinical characteristics, and treatment patterns of patients overall and stratified by age group

**Parameters at index date**	**Eligible population *N* = 2175**	**Age group**		
		Age <65, *n* = 846	Age 65–74, *n* = 759	Age ≥75, *n* = 570
**Sex, n (%)**				
Male	1169 (53.7)	439 (51.9)	412 (54.3)	318 (55.8)
Female	1006 (46.3)	407 (48.1)	347 (45.7)	252 (44.2)
**Histology, n (%)**				
Nonsquamous	1447 (66.5)	597 (70.6)	507 (66.8)	343 (60.2)
Adenocarcinoma only	1445 (66.4)	588 (69.5)	511 (67.3)	346 (60.7)
Squamous	457 (21.0)	142 (16.8)	165 (21.7)	150 (26.3)
Adenosquamous carcinoma	34 (1.6)	9 (1.1)	13 (1.7)	12 (2.1)
NSCLC NOS	152 (7.0)	61 (7.2)	49 (6.5)	42 (7.4)
Unknown	0	0	0	0
**Stage, n (%)**				
Stage 3	78 (3.6)	29 (3.4)	24 (3.2)	25 (4.4)
Stage 4	2097 (96.4)	817 (96.6)	735 (96.8)	545 (95.6)
**Number of metastatic sites, n (%)**				
0	23 (1.1)	11 (1.3)	6 (0.8)	6 (1.1)
1	1404 (64.6)	528 (62.4)	488 (64.3)	388 (68.1)
2	504 (23.2)	214 (25.3)	176 (23.2)	114 (20.0)
≥3	173 (8.0)	69 (8.2)	66 (8.7)	38 (6.7)
Missing	71 (3.3)	24 (2.8)	23 (3.0)	24 (4.2)
**Evidence of visceral metastasis**^**a**^**, n (%)**				
Present	1716 (78.9)	684 (80.9)	594 (78.3)	438 (76.8)
Absent	459 (21.1)	162 (19.1)	165 (21.7)	132 (23.2)
**Evidence of nonvisceral metastasis, n (%)**				
Present	817 (37.6)	312 (36.9)	297 (39.1)	208 (36.5)
Absent	1358 (62.4)	534 (63.1)	462 (60.9)	362 (63.5)
**Evidence of brain metastasis, n (%)**				
Present	499 (22.9)	248 (29.3)	165 (21.7)	86 (15.1)
Absent	1676 (77.1)	598 (70.7)	594 (78.3)	484 (84.9)
**Evidence of bone metastasis, n (%)**				
Present	649 (29.8)	249 (29.4)	239 (31.5)	161 (28.2)
Absent	1526 (70.2)	597 (70.6)	520 (68.5)	409 (71.8)
**Evidence of liver metastasis, n (%)**				
Present	289 (13.3)	108 (12.8)	112 (14.8)	69 (12.1)
Absent	1886 (86.7)	738 (87.2)	647 (85.2)	501 (87.9)
**ECOG PS group, n (%)**				
0 or 1	1408 (64.7)	581 (68.7)	500 (65.9)	327 (57.4)
2+	430 (19.8)	130 (15.4)	156 (20.6)	144 (25.3)
Unknown	337 (15.5)	135 (16.0)	103 (13.6)	99 (17.4)
**Line of therapy, n (%)**				
1	2175 (100.0)	846 (100.0)	759 (100.0)	570 (100.0)
2	531 (24.4)	256 (30.3)	188 (24.8)	87 (15.3)
3	136 (6.3)	73 (8.6)	43 (5.7)	20 (3.5)
≥4	38 (1.7)	25 (3.0)	7 (0.9)	6 (1.1)
**Smoking status, n (%)**				
Yes (current smoker, former smoker)	1912 (87.9)	748 (88.4)	678 (89.3)	486 (85.3)
No (never smoker)	263 (12.1)	98 (11.6)	81 (10.7)	84 (14.7)
**History of autoimmune disease, n (%)**				
Yes	33 (1.5)	12 (1.4)	13 (1.7)	8 (1.4)
No	2142 (98.5)	834 (98.6)	746 (98.3)	562 (98.6)
**Use of immunosuppressive medication, n (%)**				
Yes	385 (17.7)	133 (15.7)	132 (17.4)	120 (21.1)
No	1790 (82.3)	713 (84.3)	627 (82.6)	450 (78.9)
**Patient is immunocompromised, n (%)**^**b,c**^				
Yes	240 (11.0)	82 (9.7)	90 (11.9)	68 (11.9)
No	1935 (89.0)	764 (90.3)	669 (88.1)	502 (88.1)
**Patients with a *RET* test recorded, n (%)**	1076 (49.5)	416 (49.2)	388 (51.1)	272 (47.7)
Positive	73 (3.4)	28 (3.3)	26 (3.4)	19 (3.3)
**Patients with a *BRAF* test recorded, n (%)**	1280 (58.9)	499 (59.0)	458 (60.3)	323 (56.7)
Positive	108 (5.0)	37 (4.4)	38 (5.0)	33 (5.8)
**Patients with a *MET* exon 14 skipping mutation test, n (%)**	1062 (48.8)	409 (48.3)	385 (50.7)	268 (47.0)
Positive	0%	0	0	0
**Patients with a *RET*, *BRAF*, or *MET* test recorded, n (%)**	1307 (60.1)	509 (60.2)	466 (61.4)	332 (58.2)
Positive	179 (8.2)	65 (7.7)	62 (8.2)	52 (9.1)
**Patients with a PD-1/PD-L1 test recorded, n (%)**	1566 (72.0)	612 (72.3)	545 (71.8)	409 (71.8)
Unknown	90 (4.1)	36 (4.3)	32 (4.2)	22 (3.9)
Negative	498 (22.9)	206 (24.3)	188 (24.8)	104 (18.2)
Positive	978 (45.0)	370 (43.7)	325 (42.8)	283 (49.6)
PD-1/PD-L1 expression 1–49%^d^	62 (2.9)	23 (2.7)	17 (2.2)	22 (3.9)
PD-1/PD-L1 expression ≥50%^d^	115 (5.3)	47 (5.6)	35 (4.6)	33 (5.8)
**Patients receiving anti–PD-1/PD-L1 monotherapy, n (%)**	1103 (50.7)	379 (44.8)	361 (47.6)	363 (63.7)
Atezolizumab^e^	58 (5.3)	17 (4.5)	18 (5.0)	23 (6.3)
Nivolumab^e^	271 (24.6)	100 (26.4)	87 (24.1)	84 (23.1)
Pembrolizumab^e^	774 (70.2)	262 (69.1)	256 (70.9)	256 (70.5)
**Patients receiving anti–PD-1/PD-L1 therapy in combination with a platinum-based chemotherapy, n (%)**	989 (45.5)	447 (52.8)	357 (47.0)	185 (32.5)
Pembrolizumab + platinum-based chemotherapy^e^	950 (96.1)	429 (96.0)	344 (96.4)	177 (95.7)
**Patients receiving anti–PD-1/PD-L1 combined with any other therapy, n (%)**	83 (3.8)	20 (2.4)	41 (5.4)	22 (3.9)
Ipilimumab + nivolumab^e^	47 (56.6)	9 (45.0)	22 (53.7)	16 (72.7)

*BRAF* proto-oncogene B-Raf, *ECOG PS* Eastern Cooperative Oncology Group performance status, *HIV* human immunodeficiency virus, *MET* mesenchymal epithelial transition factor, *NOS* not otherwise specified, *NSCLC* non-small cell lung cancer, *PD-1* programmed cell death protein 1, *PD-L1* programmed cell death ligand 1, *RET* RET proto-oncogene

^a^Pulmonary (lung), hepatic (liver), pleural, pleural effusions, peritoneal and ascites involvement. Patients with visceral metastases irrespective of the presence of any other metastatic sites (eg, bone) can be categorized as visceral. All other patients without visceral metastases can be categorized as nonvisceral

^b^Immunocompromised defined as having HIV or taking long-term (≥30 days) immunosuppressive medications

^c^Having HIV, long-term use of prednisone/prednisolone, and long-term use of other immunosuppressive medications are not mutually exclusive

^d^At the time of analysis, the completeness/availability of expression results in the Patient360 NSCLC database was limited

^e^Percentages reported are for the treatment subgroup, not the eligible patient population

Only one-quarter (24.4%) of patients had recorded evidence of receiving a second line of therapy (Table 1). Of those with first-line therapy starting in 2016–2017, only 31.6% received a subsequent, second line of therapy in the study period. Among patients with first-line therapy starting between 2018 and 2020, 22.4% had subsequent treatment with a second line of therapy in the study interval.

Most patients (89.0%) were not immunocompromised, defined as having human immunodeficiency virus (HIV) or taking long-term (≥30 days) immunosuppressive medications, at the index date. Nearly all patients had a negative history of HIV, hepatitis B and C, and autoimmune disease at the index date (99.8%, 100.0%, and 98.5%, respectively). A higher percentage of female patients used immunosuppressive medication at the index date compared with male patients (20.1% versus 15.7%) (data not shown). Use of immunosuppressive medication at the index date decreased each year, with initial use at 25.5% of patients in 2016, down to 13.5% in 2020.

Overall, only 60.1% of study patients had evidence of testing for *RET-*, *BRAF-*, or *MET*-targetable genetic alterations, and 8.2% of all patients had one or more positive test results (Table 1). More patients who initiated first-line therapy between 2018 and 2020 had evidence of testing (64.2%) than those who initiated first-line therapy from 2016 to 2017 (45.6%). Almost three-quarters (72.0%) of study patients had a PD-1/PD-L1 expression test recorded, and 45.0% of study patients had a qualitative positive test result recorded. However, only 18.1% of patients with a positive PD-1/PD-L1 test had a result reported numerically. The proportion of patients tested for PD-1/PD-L1 expression increased from 53.9% in 2016 to 79.2% in 2020, and the proportion of study patients who had a positive result increased from 33.3% to 47.2% over the same period.

### Treatment patterns

**Fig. 3:**
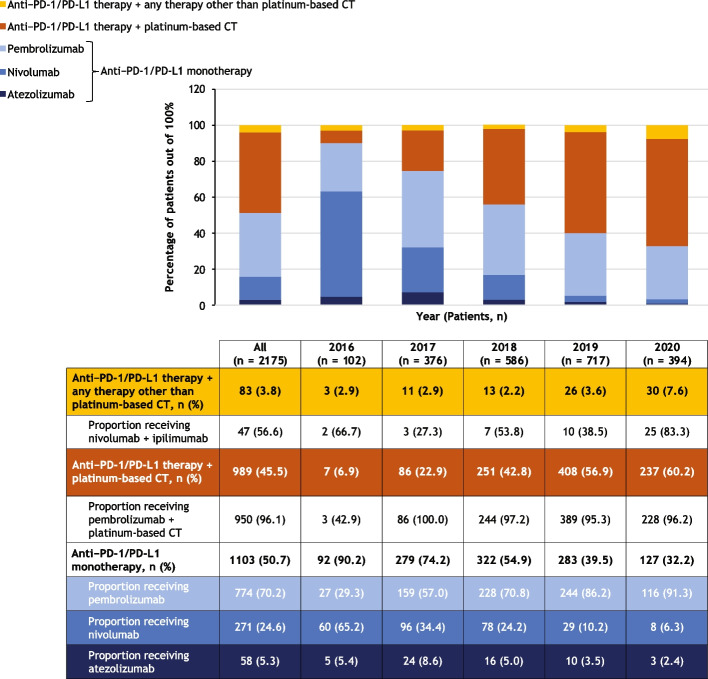
Treatment patterns overall and stratified by year of first-line therapy initiation. The graph shows the numbers of patients who received the treatments indicated in the legend. The table shows the numbers and percentages of patients who received each treatment regimen, along with proportions of patients receiving different types of anti–PD-1/PD-L1 monotherapy, pembrolizumab plus platinum-based chemotherapy, and nivolumab plus ipilimumab, which are shown below the relevant regimen category. During 2020, the COVID-19 pandemic may have impacted routine clinical care. Percentages reported for subcategories are proportions in the respective category, not the whole. CT, chemotherapy; PD-1, programmed cell death protein 1; PD-L1, programmed cell death ligand 1

Of the 2175 study cohort patients, 102 initiated first-line therapy in 2016, 376 in 2017, 586 in 2018, 717 in 2019, and 394 in 2020. First-line treatment patterns are summarized in Fig. 3.

The most common first-line treatment overall was anti–PD-1/PD-L1 monotherapy (in 50.7% of 2175 patients), followed by anti–PD-1/PD-L1 therapy in combination with a platinum-based chemotherapy (45.5%) and anti–PD-1/PD-L1 therapy in combination with any other therapy (3.8%) (Fig. 3). The proportion of patients initiating first-line treatment with anti–PD-1/PD-L1 monotherapy decreased from 90.2% of the 102 patients in 2016 to 32.2% of the 394 patients in 2020, while the proportion of patients initiating first-line anti–PD-1/PD-L1 combined with platinum-based chemotherapy increased from 6.9% to 60.2%. PD-1/PD-L1 expression levels influence treatment selection, and PD-1/PD-L1 expression data was limited at the time of the data cutoff.

Most patients who initiated treatment with an anti–PD-1/PD-L1 monotherapy in this study cohort received pembrolizumab (70.2% of the 1103 patients who received monotherapy), followed by nivolumab (24.6%), then atezolizumab (5.3%) (Fig. 3). Use of pembrolizumab increased from 29.3% of the 92 patients treated with anti–PD-1/PD-L1 monotherapy in 2016 to 91.3% of the 127 monotherapy-treated patients in 2020, whereas the use of nivolumab declined from 65.2% in 2016 to 6.3% in 2020. Pembrolizumab was consistently the most commonly used anti–PD-1/PD-L1 mono-therapy across patient subgroups including age group, sex, number of metastatic sites, evidence of brain metastasis, ECOG PS, smoking status, and immunocompromised status (Table 3). It was the most common anti–PD-1/PD-L1 monotherapy, having been administered to 73.2% of 691 patients with nonsquamous and 61.4% of 267 patients with squamous NSCLC histology.

**Table 3:** Treatment patterns by patient subgroup

**Subgroup**	n	**Anti–PD-1/ PD-L1 monotherapy, n (%)**	Atezolizumab, n (%)	Nivolumab, n (%)	Pembrolizumab, n (%)	**Anti–PD-1/ PD-L1 therapy + platinum-based CT, n (%)**	Pembrolizumab + platinum-based CT, n (%)	**Anti–PD-1/ PD-L1 therapy + any therapy other than platinum-based CT, n (%)**	Nivolumab + ipilimumab, n (%)
**Age group**									
<65 years	846	**379 (44.8)**	17 (4.5)	100 (26.4)	262 (69.1)	**447 (52.8)**	429 (96.0)	**20 (2.4)**	9 (45.0)
65–74 years	759	**361 (47.6)**	18 (5.0)	87 (24.1)	256 (70.9)	**357 (47.0)**	344 (96.4)	**41 (5.4)**	22 (53.7)
≥75 years	570	**363 (63.7)**	23 (6.3)	84 (23.1)	256 (70.5)	**185 (32.5)**	177 (95.7)	**22 (3.9)**	16 (72.7)
**Sex**									
Male	1169	**581 (49.7)**	36 (6.2)	137 (23.6)	408 (70.2)	**548 (46.9)**	526 (96.0)	**40 (3.4)**	23 (57.5)
Female	1006	**522 (51.9)**	22 (4.2)	134 (25.7)	366 (70.1)	**441 (43.8)**	424 (96.1)	**43 (4.3)**	24 (55.8)
**Histology**									
Nonsquamous	1447	**691 (47.8)**	31 (4.5)	154 (22.3)	506 (73.2)	**705 (48.7)**	686 (97.3)	**51 (3.5)**	25 (49.0)
Squamous	457	**267 (58.4)**	19 (7.1)	84 (31.5)	164 (61.4)	**168 (36.8)**	164 (97.6)	**22 (4.8)**	18 (81.8)
**Number of metastatic sites**									
0	23	**12 (52.2)**	1 (8.3)	5 (41.7)	6 (50.0)	**9 (39.1)**	9 (100.0)	**2 (8.7)**	2 (100.0)
1	1404	**734 (52.3)**	42 (5.7)	189 (25.7)	503 (68.5)	**616 (43.9)**	592 (96.1)	**54 (3.8)**	33 (61.1)
2	504	**243 (48.2)**	9 (3.7)	46 (18.9)	188 (77.4)	**238 (47.2)**	230 (96.6)	**23 (4.6)**	11 (47.8)
≥3	173	**73 (42.2)**	1 (1.4)	19 (26.0)	53 (72.6)	**97 (56.1)**	91 (93.8)	**3 (1.7)**	0
**NSCLC stage at index date**									
3	78	**42 (53.8)**	4 (9.5)	12 (28.6)	26 (61.9)	**33 (42.3)**	32 (97.0)	**3 (3.8)**	2 (66.7)
4	2097	**1061 (50.6)**	54 (5.1)	259 (24.4)	748 (70.5)	**956 (45.6)**	918 (96.0)	**80 (3.8)**	45 (56.3)
**Brain metastases**									
Yes	499	**261 (52.3)**	9 (3.4)	50 (19.2)	202 (77.4)	**221 (44.3)**	210 (95.0)	**17 (3.4)**	8 (47.1)
No	1676	**842 (50.2)**	49 (5.8)	221 (26.2)	572 (67.9)	**768 (45.8)**	740 (96.4)	**66 (3.9)**	39 (59.1)
**ECOG PS**									
0 or 1	1408	**653 (46.4)**	26 (4.0)	152 (23.3)	475 (72.7)	**698 (49.6)**	670 (96.0)	**57 (4.0)**	35 (61.4)
≥2	430	**242 (56.3)**	8 (3.3)	61 (25.2)	173 (71.5)	**171 (39.8)**	163 (95.3)	**17 (4.0)**	7 (41.2)
**Smoking status**									
Current or former	1912	**946 (49.5)**	45 (4.8)	226 (23.9)	675 (71.4)	**897 (46.9)**	860 (95.9)	**69 (3.6)**	41 (59.4)
Never	263	**157 (59.7)**	13 (8.3)	45 (28.7)	99 (63.1)	**92 (35.0)**	90 (97.8)	**14 (5.3)**	6 (42.9)
**Patient immunocompromised**^**a, b**^									
Yes	240	**126 (52.5)**	9 (7.1)	37 (29.4)	80 (63.5)	**95 (39.6)**	86 (90.5)	**19 (7.9)**	11 (57.9)
**History of autoimmune disease**									
Yes	33	**11 (33.3)**	1 (9.1)	4 (36.4)	6 (54.5)	**15 (45.5)**	14 (93.3)	**7 (21.2)**	5 (71.4)
No	2142	**1092 (51.0)**	57 (5.2)	267 (24.5)	768 (70.3)	**974 (45.5)**	936 (96.1)	**76 (3.5)**	42 (55.3)
**Patients positive for *RET*, *BRAF*, or* MET* mutations**									
Yes	179	**94 (52.5)**	1 (1.1)	20 (21.3)	73 (77.7)	**77 (43.0)**	75 (97.4)	**8 (4.5)**	4 (50.0)
**PD-1/PD-L1 expression**^**c**^									
1–49%	62	**27 (43.5)**	5 (18.5)	10 (37.0)	12 (44.4)	**35 (56.5)**	35 (100.0)	**0**	0
≥50%	115	**84 (73.0)**	1 (1.2)	6 (7.1)	77 (91.7)	**26 (22.6)**	23 (88.5)	**5 (4.3)**	0

*BRAF* proto-oncogene B-Raf, *CT* chemotherapy, *ECOG PS* Eastern Cooperative Oncology Group performance status, *HIV* human immunodeficiency virus, *MET* mesenchymal epithelial transition factor, *NSCLC* non-small cell lung cancer, *PD-1* programmed cell death protein 1, *PD-L1* programmed cell death ligand 1, *RET* RET proto-oncogene

^a^Immunocompromised defined as having HIV or taking long-term (≥30 days) immunosuppressive medications

^b^Having HIV, long-term use of prednisone/prednisolone, and long-term use of other immunosuppressive medications are not mutually exclusive

^c^At the time of analysis, the completeness/availability of expression results in the Patient360 NSCLC database was limited

Pembrolizumab combined with platinum-based chemo-therapy was the most common combination regimen, accounting for 96.1% of the 989 study cohort patients who initiated first-line treatment with anti–PD-1/PD-L1 therapy combined with platinum-based chemotherapy (Fig. 3). Of the 83 patients (3.8% of the cohort) who initiated first-line treatment with anti–PD-1/PD-L1 therapy in combination with any other therapy, 56.6% received nivolumab plus ipilimumab.

From 2018 to 2020, the most common first-line treatment among patients younger than 65 years was anti–PD-1/PD-L1 in combination with platinum-based chemotherapy (61.1%); and in those 75 years or older, the most common regimen was anti–PD-1/PD-L1 monotherapy (56.7%) (data not shown). During the same period, 55.3% of patients with nonsquamous NSCLC were treated with anti–PD-1/PD-L1 in combination with platinum-based chemotherapy, and 40.8% received anti–PD-1/PD-L1 monotherapy (data not shown). The proportions of patients with 1, 2, or ≥3 metastatic sites treated with anti–PD-1/PD-L1 in combination with platinum-based chemotherapy were 50.9%, 54.6%, and 64.7%, respectively (data not shown). From 2018 to 2020, anti–PD-1/PD-L1 in combination with platinum-based chemotherapy was also the most commonly used regimen in patients with ECOG PS 0 or 1 (55.5%), current or former smokers (53.6%), and those who were not immunocompromised (53.9%) (data not shown).

## Discussion

This retrospective, real-world cohort study using the ConcertAI Patient360 NSCLC database demonstrated that, from 2016 to 2020, the most common first-line treatment among US patients with locally advanced or metastatic NSCLC who received IO treatment was anti–PD-1/PD-L1 monotherapy, followed by anti–PD-1/PD-L1 agents combined with platinum-based chemotherapy. Nivolumab was the most common monotherapy in 2016, but after the negative CheckMate-026 trial, was overtaken by pembrolizumab in 2017, which remained the most frequently used monotherapy agent until the end of the study period in 2020, corresponding with the positive KEYNOTE-024 trial results [15, 20]. A shift in the most commonly used treatments occurred during the study period, from predominantly anti–PD-1/PD-L1 monotherapy in 2016 to combination treatment with anti–PD-1/PD-L1 agents and platinum-based chemotherapy. This was likely driven by US regulatory approval of IO-chemotherapy combination regimens and positive clinical trial results after earlier approvals of IO monotherapies [21–25].

Several patient characteristics in this real-world cohort differed from those in pivotal IO clinical trials [5, 9, 11, 12]. The median age of 68.0 years was numerically higher than the median of approximately 60–65 years in several clinical trials [5, 9, 11, 12, 26], and 26.2% of the patients in this study were 75 years or older. This finding is consistent with another real-world study [26], which demonstrated that patients receiving IO in the clinic are substantially older than patients studied in the trials that led to these agents’ approvals, as NSCLC trials often recruit fewer elderly patients [26]. Clinical trials also typically exclude patients with ECOG PS >1, but almost 20% of the patients in this study had ECOG PS ≥2. In our study, ECOG PS deteriorated with age, suggesting that including greater proportions of elderly patients may make clinical trials substantially more generalizable to the real-world setting. Compared with real‑world studies of patients receiving first-line treatment for metastatic NSCLC [18, 27], our study included a higher proportion of patients with an ECOG score ≥2 and a higher proportion of patients with brain metastases.

Patients with a history of autoimmune disease and those who are immunocompromised or receiving immuno-suppressive medications are also generally excluded from clinical trials of IO, but these types of patients comprised 1.5%, 11.0%, and 17.7%, respectively, of this real-world cohort and received first-line IO therapy for NSCLC. The fact that 78.9% of this study cohort had visceral metastases and 37.6% had nonvisceral metastases suggests that a substantial proportion of this cohort had dual visceral and nonvisceral metastases.

This study has several strengths and limitations. The database provided valuable real-world data on diagnosis, clinical assessment, and recorded treatments in patient groups not typically enrolled in clinical trials, such as older patients and those with higher ECOG PS, immuno-suppression, and various types and numbers of metastatic sites.

This was a descriptive observational study (no hypothesis testing) and has several limitations. The study may have been subject to confounding if physicians preferentially prescribed certain therapies to patients who were perceived to have worse adverse effects if their underlying disease was more severe or if they had poorer overall health. The study was not designed to evaluate patient outcomes; therefore, no conclusions on prognosis may be drawn. As with all retrospective epidemiological studies, unmeasured confounding and missing data may have an impact on the descriptive estimates presented.

Data entry errors at the points of care could not be detected nor corrected during analysis. Missing data in the form of information not routinely and repeatedly captured may also have impacted the completeness, validity, and reliability of some variables (eg, PD-L1 testing). Potentially interesting data that were unavailable for analysis included the rate of transition to second-line treatment when stratified by first-line therapy and information on local radiotherapy. This study included patients who were treated during the COVID-19 pandemic, which may have impacted routine clinical care in the year 2020. Finally, patients treated at individual sites included in this study may not be representative of all patients with NSCLC across all the sites of care in the US. This study highlights the differences in patient characteristics between real-world populations and clinical trial populations, presenting difficulties in treating patients underrepresented in clinical trial populations. Incorporating more diverse, traditionally excluded patient populations will increase the generalizability of studies and provide the evidence-base required to support decision-making in routine clinical practice.

## Conclusions

The initial adoption of anti–PD-1/PD-L1 monotherapy as first-line treatment for advanced NSCLC in the US quickly shifted to combination anti–PD-1/PD-L1 therapy with platinum-based chemotherapy between 2016 and 2020. This real-world study was conducted during these important inflection points for the treatment of advanced NSCLC with anti–PD-1/PD-L1 therapy. This study has emphasized the real-world patient characteristics, how they differ from clinical trial populations, and how these characteristics impact treatment patterns.

In conclusion, we have shown that evidence gaps exist for patients who are older, have ECOG PS ≥2, and are on immunosuppressive medications—patients who make up a substantial proportion of real-world patient populations. To optimize the personalized treatment of advanced NSCLC, further real-world studies will be needed to elucidate the clinical characteristics of patients with advanced NSCLC who are most likely to benefit from an evolving first-line IO treatment landscape.

## Data Availability

The data that support the findings of this study are available from ConcertAI but restrictions apply to the availability of these data, which were used under license for the current study, and so are not publicly available. The corresponding author may be contacted regarding potential access to the data upon reasonable request and with permission of ConcertAI.

## Abbreviations

1L: First line
*ALK*: Anaplastic lymphoma kinase
*BRAF*: Proto-oncogene B-Raf
CT: Chemotherapy
CTLA-4: Cytotoxic T lymphocyte-associated antigen 4
ECOG PS: Eastern Cooperative Oncology Group performance status
*EGFR*: Epidermal growth factor receptor
EMR: Electronic medical record
FDA: Food and Drug Administration
HIV: Human immunodeficiency virus
IO: Immunotherapy
*MET*: Mesenchymal epithelial transition factor
NOS: Not otherwise specified
NSCLC: Non-small cell lung cancer
PD-1: Programmed cell death protein 1
PD-L1: Programmed cell death ligand 1
*RET*: RET proto-oncogene
*ROS1*: ROS proto-oncogene 1
US: United States
